# Supplementary material for: Identification and expression analysis of xyloglucan endotransglucosylase/hydrolase (XTH) family in grapevine (Vitis vinifera L.)
Source: PeerJ. 2022 Jun 13;10:e13546. doi: 10.7717/peerj.13546 (PMC9202548; doi:10.7717/peerj.13546)
Supplement: Supplemental Information 1 [file peerj-10-13546-s001.zip › RAW DATA/multiple sequence alignments/multiple sequence alignments.pdf]

```
VvXTH01/1-280  ...........................................................................................
VvXTH02/1-314  .........................................................................MA.....LLQQKLLASLL
VvXTH03/1-308  ..............................................MLTTLP.............QSLPLGTKMASCL....WL..LSLGL
VvXTH04/1-342  .........................................................................MG.....QLPVIRNFSLF
VvXTH05/1-295  ............................................................MNDYHKLV.........F.........FIGV
VvXTH06/1-282  .............................................................................M....GS..SSNGY
VvXTH07/1-280  .............................................................................M....GF..SSYEY
VvXTH08/1-282  .............................................................................M....GS..SSNGY
VvXTH09/1-282  .............................................................................M....GS..SSNRY
VvXTH10/1-292  .........................................................................MA..........PLLCLL
VvXTH11/1-281  .........................................................................MAS............LLA
VvXTH12/1-294  .........................................................................MA..........FFIFLV
VvXTH13/1-301  .........................................................................ME..L....QA..SPVPI
VvXTH14/1-295  ..........................................................................MEAF....SS..PKVSS
VvXTH15/1-291  .........................................................................MAPCF....SL..NISSM
VvXTH16/1-292  ...................................................................................M..GSFQV
VvXTH17/1-322  ...........................................................................................
VvXTH18/1-370  MLKFDEWCLGNGKVDLHFSLLNPCKIRLLTEPSSRSPSTFPHDTYYKTPTYPRHSLHFHAQNYFHFSSLNSHSMASPS....TW..NLSPK
VvXTH19/1-298  .................................................................MAPIC.......S....SS..SAVST
VvXTH20/1-286  ........................................................................................MAS
VvXTH21/1-279  ...........................................................................................
VvXTH22/1-313  ..................................................MPTKCSIIPPQLKYHFQVSVTYSMAS.S....PF..SSSSL
VvXTH23/1-297  .........................................................................MASPS....TS..NLSPK
VvXTH24/1-298  .................................................................MAPIY.......S....SS..SAVST
VvXTH25/1-297  .........................................................................MASPS....TS..NLSPK
VvXTH26/1-297  .........................................................................MTYPT....TS..NLSPK
VvXTH27/1-270  .................................................................MAPIY.......S....SS..SAVST
VvXTH28/1-297  .........................................................................MASTS....TS..NLSPK
VvXTH29/1-263  ...........................................................................................
VvXTH30/1-273  .................................................................MAPIY.......S....SS..PAVPT
VvXTH31/1-297  .........................................................................MAVPS.........VLPL
VvXTH32/1-323  .........................................................................MAKS........LFICSI
VvXTH33/1-252  .......................................................................MVMEYSLVDLIRHVIQVGTM
VvXTH34/1-285  ........................................................................................MST

                                                                          1       10        20        30
VvXTH01/1-280  ...........................................................MSLDERTGSGFISQDLYLHGFFSASIKLPAD.
VvXTH02/1-314  VIF...GISAAVSAGNGHDAAPNVTRLTDLFGHLTFNHGFTEFFGGSNIQPINNGSYANLILNKSSGSGLVSQSKYYYGFFSAAIKLPSG.
VvXTH03/1-308  L.FMVSGAR....G...........AAPRKPIDVPFGRNYVPTWAFDHIKSYNGGSEIQLVLDKYTGTGFQSKGSYLFGHFSMQIKMVPG.
VvXTH04/1-342  FFF...FVSFFCA............ASSFNLSVISFDQGYTHLFGEGNLVRSSDGRSVRLLLDRYTGSGFISANLYNHGFFSANIKLPSE.
VvXTH05/1-295  L.TLISLVQ....V...........SDASVVSTGDFNKDFFVIWSPSHVNTSADGHARSLKLDKESGAGFASNQLFLFGQIDMQIKLVTG.
VvXTH06/1-282  S.VFLLLFA....V............VVATASASNFYQDFDLTWGDNRAKIFNGGQLLSLSLDQASGSGFQSKKEYLFGRIDMQLKLVAG.
VvXTH07/1-280  S.VLVLVLA....M............MVATASASNFYQDFDITWGDHRAKMFNGGQLLSLSLDKVSGSGFQSKREYLFGRIDMQLKLVAG.
VvXTH08/1-282  S.FFGLVFA....M............VLATASASNFYQDFDITWGDHRAKIFNGGQLLSLSLDKTSGSGFQSKKEYLFGRIDMQLKLVAG.
VvXTH09/1-282  S.FFCLVFA....M............VLATASAGNFYQDFDITWGDHRAKIFNGGQLLSLSLDKTSGSGFQSKKEYLFGRIDMQLKLVAG.
VvXTH10/1-292  ILM.....ITTCSDAQGPPSPGF..SPSSKFSPISFNQGFRNLWGSQHQ..TIDQGLLTIWLDSTSGSGFKSLKPYRSGYFSAAIKLQPG.
VvXTH11/1-281  L.LLAFLLLG...A...........VVGTKMSDITFDQSYRITWGNDHVLSLNQGRLVQLSLDQSSGAGFASKQSYGSGFFHMRIKLPPK.
VvXTH12/1-294  LVL.....MVPSSNAQWPPSPGY..YPSRKFRSMSFYQGFRNLWGPQHQ..RVDQNALTIWLDSTSGSGFKSVRPFRSGYFGASIKLQPG.
VvXTH13/1-301  LLCIAALMAASCPP...........SQAEGQSKSSFEDSFDIMWSEDHFKTSEDGQIWYLSLDKETGCGFQTKQRYRFGWFSMKLKLVGG.
VvXTH14/1-295  L.LFSLFVS....S............LMATASAGNFYQDFDLTWGDRRAKIVDGGKLLMLSLDRASGSGFQSKKEYLFGRIDMQLKLVSG.
VvXTH15/1-291  L.LFSVFVS....S............IMA.ASAGSFYQDFDLTWGDHRAKILDGEKLLTLSLDKASGSGFQSKKEYLFGKIDMQLKLVSG.
VvXTH16/1-292  L.FVALLVTA...V...........AFEQSFVDANFSKSMYFNWGASHSSILGNGDDLQLVLDSTSGSGVQTKRAFLFGSIEMLIKLVPG.
VvXTH17/1-322  ............................MAASAGNFYQDFDITWGDGRAKILNNGELLTLSLDKTSGSGFQSKNEYLFGKIDMQLKLVPG.
VvXTH18/1-370  L.FLVSLLL....G............SFVVASVGNLYQDFEITWGDGRAKILNNGELLTLSLDKTSGSGFQSKNEYLFGKIDMQLKLVPG.
VvXTH19/1-298  A.LLMSVVV....S............FLMAASAGNFYQDFDITWGDGRGKILNNGELLTLSLDKPSGSGFQSKNEYLFGKIDMQLKLVPG.
VvXTH20/1-286  S.SILVLML....S............MLVVASGGNFNQDFDITWGDGRAKILNNGQLLTLSLDKASGSGFQSRNEYLFGKIDMQLKLVSG.
VvXTH21/1-279  .......MA....A............SFIAVSAGTLYEDIDIKWGDDRAKILNNGQLLTLSLDRTCGAGFQSKNEYLFGKIEMQLKLVPG.
VvXTH22/1-313  L.GLVVYMF....C............SLMAASAGNFYQEFDITWGNDRAKLLDKGQLLTLSLDRASGSGFKSKNEYLFGKIDMQLKLVPG.
VvXTH23/1-297  L.LLLSLLF....G............SFVVASVGNLYQDLDITWGDGRAKILDNGQLLTLSLDKASGSGFQSQDEYLFGKIDMQLKLVPG.
VvXTH24/1-298  A.LLISVVV....S............FLMAASAGNFYQDFGITWGDGRAKILDNGEFLTLSLDKTSGSGFQSKNEYLFGKIDMQLKLVPG.
VvXTH25/1-297  L.LLMSLLL....G............SFVVASVGNLYQDFDLTWGDGRAKILNNGQLLTLSLDKASGSGFQSKNEYLFGKIDMQLKLVPG.
VvXTH26/1-297  L.LLLSLLL....G............SFVVASVGNLYQDFDITWGDGRAKILNNGQLLTLSLDKASGSGFRSNNEYLFGKIDMQLKLVPG.
VvXTH27/1-270  A.LLISVVV....S............FLMAASAGNFYQDFDITWGDRRAKILDNGEFLTLSLDTTSGSGFQSKNEYLFGKIDMQLKLVPG.
VvXTH28/1-297  L.LLLSLLL....G............SFVVASVGNLYQDFDLTWGDGRAKILNNGQLLTLSLDKASGSGFQSKNEYLFGKIDMQLKLVPG.
VvXTH29/1-263  .......................................MLFSFLTFLAKILDNGQLLTLSLDKTSGSGFQSKNEYMFGKIDMQLKLVPG.
VvXTH30/1-273  A.MHISVVV....S............FLMAASAGNFNQDFDITWGDGRAKILNSGELLTLSLDKTSGSGFQSKNEYLFGKIDMQLKLVPG.
VvXTH31/1-297  L.FGFLAFSV...V...........VFVGSASSSKFTELFQPGWAFDHF..LYEGELLKLKLDNYSGAGFSSKSKYMFGKVSIQIKLVEG.
VvXTH32/1-323  LLLLNSFLSFRSSATNLAYSTPF...DRWFFKVFGDEN..........IKSVHNGTVAKISLDQKTGSEFVSSDIFLHGYFSASIKLPRNQ
VvXTH33/1-252  IRFLVKSLDEEILQISGSLNPPH...TGSLFMIFCSSQSLAARNGTSPFPYFATTRNLEISLDQKTGSELVSSNIFLHGYFSASIKLPRSQ
VvXTH34/1-285  L.FLVALMAL...A...........IVQNSAMSDDFNQLFQPNWAPDHI..STEGDQIKLTLDTISGCGFESKKKYLFGKVSMQVKLVEG.
```

```
                40        50        60        70        80        90        100       110
VvXTH01/1-280  YTAGVVVAFYMSNADMFEANHDELDFEFLGNIRGKDWRIQTNVYGNG....STNVGREERYHLWFDPSEDFHQYSILWSDNLIIFYVDNVP
VvXTH02/1-314  YTSGVVVAFYMSNADLFPHNHDEIDIELLGHEKRKDWVLQTNMYGNG....SVSTGREEKFRLWFDPTEQYHHYSFIWNRHHIVFLVDNVP
VvXTH03/1-308  DSAGTVTAFYLSS...QNSEHDEIDFEFLGNRSGQPYILQTNVFTGGKG......DREQRIYLWFDPTKAFHSYSVLWNTFLIIFLVDDVP
VvXTH04/1-342  YTAGVVVAFYTSNGDVFEKTHDELDFEFLGNVKGKPWRFQTNVYGNG....STSRGREERYRLWFDPSKEFHRYSILWTAKNIIFYVDEVP
VvXTH05/1-295  HSAGTVVAYYLTS...DQPNRDEVDFEFLGNVSGQPYILQTNVFADGFD......NREERIYLWFDPTEDFHTYSILWNLHQIVFMVDWVP
VvXTH06/1-282  NSAGTVTAYYLSS...QGSTHDEIDFEFLGNLSGDPYILHTNVFTQGKG......NREQQFYLWFDPTRNFHTYSIIWTARHIIFLVDNVP
VvXTH07/1-280  NSAGTVTAYYLSS...QGPTHDEIDFEFLGNLSGDPYILHTNMFTQGKG......NREQQFYLWFDPTRNFHTYSVIWNPQQIIFLVDNTP
VvXTH08/1-282  NSAGTVTAYYLSS...QGPTHDEIDFEFLGNLSGDPYILHTNVFTQGKG......NREQQFYLWFDPTRNFHTYSIAWSAQHIIFLVDNVP
VvXTH09/1-282  NSAGTVTAYYLSS...QGPTHDEIDFEFLGNLSGDPYILHTNVFTQGKG......NREQQFYLWFDPTRNFHTYSIAWSAQHIIFLVDNVP
VvXTH10/1-292  YTAGVITSFYLSNNEDYPGNHDEIDIEFLGTTLDKPYTLQTNVYMKGSG.DGRLIGREVKIHLWFDPTEDFHNYAILWTPDEIIFLVDDVP
VvXTH11/1-281  DSAGVVTAYYLTS...HTKNHDELDFEFLGNKEGKPYTLQTNVFSNGQG......DREQRILLWFDPTADFHSYKILWNQHQIVFYVDDIP
VvXTH12/1-294  YTAGVITAFYLSNNEAHPGFHDEVDIEFLGTTFGKPYTLQTNVYIRGSG.DGRIIGREMKFHLWFDPTKDFHHYAILWSPKDIIFFVDDVP
VvXTH13/1-301  DSAGVVTAYYMCSENGAGPERDELDIEFLGNRSGEPYLIQTNVYKNGTG......GREMRHMLWFDPTEEFHSYSILWNNEQILFVVDRVP
VvXTH14/1-295  NSAGTVTAYYLSS...QGQTHDEIDFEFLGNLSGDPYILHTNVFTQGKG......NREQQFYLWFDPTKNFHTYSIIWNPQRIIFLVDNFP
VvXTH15/1-291  NSAGTVTAYYLSS...QGPTHDEIDFEFLGNLSGDPYILHTNVFTQGKG......NREQQFYLWFDPTENFHTYSIIWNLQNIIFLVDNIP
VvXTH16/1-292  NSAGTVTAYYLSS...TGSKHDEIDFEFLGNVSGQPYTIHTNIYTQGNG......SREQQFYLWFDPTADFHNYTIHWNPTEVVWYIDSIP
VvXTH17/1-322  NSAGTVTAYYLSS...QGPTHDEIDFEFLGNLSGDPYILHTNVFSQGKG......NREQQFYLWFDPTADFHTYSILWNPQRIIFSVDGTP
VvXTH18/1-370  NSAGTVTAYYLSS...QGPTHDEIDFEFLGNLSGDPYILHTNVFSQGKG......NREQQFYLWFDPTADFHTYSILWNPQRIIFSVDGTP
VvXTH19/1-298  NSAGTVTAYYLSS...QGPTHDEIDFEFLGNLSGDPYILHTNVFSQGKG......NREQQFYLWFDPTADFHTYSILWNPQRIIFSVDGTP
VvXTH20/1-286  NSAGTVTAYYLSS...QGSAHDEIDFEFLGNLSGDPYILHTNVFSQGKG......NREQQFYLWFDPTADFHTYSILWNPQLIIFSVDGTP
VvXTH21/1-279  NSAGTVTAFYLSS...PGPTRDEIDFEFLGNLSGDPYTVHTNVYTQGKG......EREQQFHLWFDPTADFHTYSFTWNPKHIIFSVDDTP
VvXTH22/1-313  NSAGTVTAYYFSS...LGSTHDEIDFEFLGNLSGDPYILHTNVFSQGKG......NREQQFYLWFDPTANFHTYSVLWNPHNILFSVDGTP
VvXTH23/1-297  NSAGTVTAYYLSS...QGPTHDEIDFEFLGNLSGDPYILHTNVFSQGKG......NREQQFYLWFDPTADFHTYSVLWNPQRIIFSVDGTP
VvXTH24/1-298  NSAGTVTAYYLSS...QGPTHDEIDFEFLGNLSGDPYILHTNVFSQGKG......NREQQFYLWFDPTADFHTYSILWNPQRIIFSVDGTP
VvXTH25/1-297  NSAGTVTAYYLSS...QGPTHDEIDFEFLGNLSGDPYILHTNVFSQGKG......NREQQFYLWFDPTADFHTYSILWNPQRIIFSVDGTP
VvXTH26/1-297  NSAGTVTAYYLSS...QGPTHDEIDFEFLGNLSGDPYILHTNVFSQGKG......NREQQFYLWFDPTADFHTYSVLWNPQRIIFSVDGTP
VvXTH27/1-270  NSAGTVTAYYLSS...QGPTHDEIDFEFLGNLSGDPYILHTNVFSQGKG......NREQQFYLWFDPTADFHTYSILWNPQRIIFSVDGTP
VvXTH28/1-297  NSAGTVTAYYLSS...QGPTHDEIDFEFLGNLSGDPYILHTNVFSQGKG......NREQQFYLWFDPTADFHTYSILWNPQRIIFSVDGTP
VvXTH29/1-263  NSAGTVTAYYLSS...QGPTHDEIDFEFLGNLSGDPYILHTNVFSQGKG......NREQQFYLWFDPTANFHSYSILWNPQRIIFSVDGTP
VvXTH30/1-273  NSAGTVTAYYLSS...QGPTHDEIDFEFLGNLSGDPYILHTNVFGQGKG......NRE.........................HFSVDGTP
VvXTH31/1-297  DSAGTVTAFYMSS...DGPNHNEFDFEFLGNTTGEPYLVQTNIYVNGVG......NREQRLNLWFDPTKDFHSYTFMWNQRQVVFLVDETP
VvXTH32/1-323  DTAGVVVAFFTMNQDIYSRNQDEATFEFLGNVKGKEWVVQTNIHGNTTIRNSTSKGREERYTLWFDPSADFHKYSILWTENHIIFYVDDVP
VvXTH33/1-252  GTVGVVVAFDSMNQDRYSKNQDEVTFEILGNVKGKEWAVQTNVHGNTTIRNSTSKGREERYTLRFDPSADFHKYSIFWTENHIIFYVDDVP
VvXTH34/1-285  DSAGTVTALYMAS...EGLNHDELDFEFLGNVSGEPYLVQTNVYVNGTG......NREQRHTLWFDPTAAFHTYSFLWNRYSILFLVDEVP

               120       130       140       150       160       170       180       190
VvXTH01/1-280  IREIKRTEAMGGDFPS.KPMSLYATIWDGSEWATNGGRYRVNYKYAPYVAEFSNLVL.HGCAVDPIEKSP..K..CDNG.P....TSKASI
VvXTH02/1-314  VREILYNDAISSVYPS.KPMSVYATIWDGSEWATHGGKYPVDYKYAPFVVSFGEMEM.DGCTFDPKKKGV..S..CSKGSVSSRDPVDGEE
VvXTH03/1-308  IRVFKNSKDLGVKFPFDQPMKIYSSLWNADDWATRGGLEKTDWSKAPFVASYKSFHV.DGCEASVEAK......FCATQGKRW...WDQRE
VvXTH04/1-342  IREVIRNEAMGGDYPS.KPMALYATIWDASNWATSGGKYKVDYNYAPFVSEFSDFVL.DGCPADPLQLAS..AGGCSDKDA....ELESND
VvXTH05/1-295  IRVYRNHADKGVAFPRWQPMSIKISLWNGDSWATRGGKDKIDWTKGPFIASFRNYKL.DACIWRGNAR......FCRADSPT..NWWNKER
VvXTH06/1-282  IRLFKNAESMGVPFPKNQPMRIYSSLWNADDWATRGGLVKTDWSKAPFTAYYRNFRA.NS................STPTS.SFPDSTFQ
VvXTH07/1-280  IRLFRNAESMGVPFPKNQPMRIYSSLWNADDWATRGGLVKTDWSKAPFTAYYRNFRA.YT................STSSN.SFSNSAWQ
VvXTH08/1-282  IRLFKNAESMGVPFPKNQPMRIYSSLWNADDWATRGGLVKTDWSKAPFTAYYRNFRA.ST................STSTS.TFSDSAFQ
VvXTH09/1-282  IRLFKNAESMGVPFPKNQPMRIYSSLWNADDWATRGGLVKTDWSKAPFTAYYRNFRA.ST................STSTS.TFSDSAFQ
VvXTH10/1-292  VRRYPRKND..ATFPA.RPMWVYGSIWDASSWATEDGKYKADYRYQPFIGRYKNFKL.GGCTTDAAAS.......CRPPS......ISPSS
VvXTH11/1-281  IRVFKNNSNIGVDYPS.QPMQIEGSLWNGESWATDGGQTKTNWSCAPFRTHFQGFRI.DGCPSDYS........NC.....HSSNLWWNQKK
VvXTH12/1-294  VRRYPRKSA..TTFPL.RPMWLYGSIWDASSWATEDGKYKADYRYQPFVGQYSNFKA.SGCSAYSPAS.......CRPVS......ASPYR
VvXTH13/1-301  IRVYKNNGKANNFFPNEKPMYLFSSIWNADDWATRGGMEKTDWKKAPFVSSYKDFSV.DGCQWEDPYP......ACVSTTTQ..NWWDQYE
VvXTH14/1-295  IRQFQNQESIGIPFPKSQPMRIYSSLWNADNWATRGGVVKIDWSKAPFTAFYRNFNA.AACIWSYGSS......SCASKSVS.PMSNIGWQ
VvXTH15/1-291  IREFKNQESIGVPFPKSQPMRIYSSLWNADDWATRGGLVKIDWSKAPFTASYRNFNA.AACLWSHGSS......SCASKFTS.PVSNIAWQ
VvXTH16/1-292  IRVFRNYENEGIAYPSKQGMRVYSSIWNADNWATRGGLVKIDWYSAPFVARFRHFRA.RACKWNGPVSID....QCASKSPA..NWWTSPV
VvXTH17/1-322  IREFKNSESIGVSYPKNQPMRIYSSLWNADDWATRGGLVKTDWSQAPFTASYRNFNA.DACIWSSGAS......SCSSNTPTSTSTNTEWY
VvXTH18/1-370  IREFKNSESIGVSYPKNQPMRIYSSLWNADDWATRGGLVKTDWSQAPFTASYRNFNA.DACIWSSGAS......SCSSTTPSSTSTSGQWY
VvXTH19/1-298  IREFKNSESIGVPYPKNQPMRIYSSLWNADDWATRGGLVKTDWSQAPFTASYRNFNA.NACLWSSGSS......SCSSTSPSSTSTNGGWY
VvXTH20/1-286  IRQFKNLESKGVAYPKNQPMRIYSSLWNADDWATRGGLVKTDWSQAPFTASYRNFNA.NACVWSSGSS......SCSKNSSS..TTSNAWL
VvXTH21/1-279  IREFKNLESIGVQYPKNQAMSLYSSLWDAEDWATRGGLVKTDWSQAPFTASYRNFNA.NGCIWSAGKS......SCSSVSSSLILADNSWL
VvXTH22/1-313  IRVFKNLEAMGIPFPKKQPMRIYSSLWNADDWATRGGLVKTDWSQAPFTASYRNFDASNACVWSSGAS......SCSSKSPS..SSNSPWM
VvXTH23/1-297  IREFKNSESIGVPYPKNQPMRIYSSLWNADDWATRGGLVKTDWSLAPFTASYRNFNA.NACVWSSGSS......SCSSNSPSSTSTNGGWY
VvXTH24/1-298  IREFKNSESIGVPYLKNQPMRIYSSLWNADDWATRGGLIKTDWTQAPFTASYRNFNA.DACIWSSGAS......SCSSNTPTSISPSTDWY
VvXTH25/1-297  IREFKNSESIGVPFPKNQPMRIYSSLWNADDWATRGGLVKTDWSQAPFTASYRNFNA.NACVWSSGSS......SCSSTSPSSTSTNSGWY
VvXTH26/1-297  IREFKNSESIGVPYPKNQPMRIYSSLWNADDWATRGGLVKTDWSQAPFTASYRNFNA.NACVWSSGSS......SCSSTSPSSTSTNGGWY
VvXTH27/1-270  IREFKNSESIGVPFPKNQPMRIYSSLWNADDWATRGGLVKTDWTQAPFTASYRNFNA.DACIWSSGAF......SCSSNTPLLSPPTHQHS
VvXTH28/1-297  IREFKNSESIGVPFPKNQPMRIYSSLWNADDWATRGGLVKTDWTQAPFTASYRNFNA.NACVWSSRSS......SCSSTSPSSTSTNGGWY
VvXTH29/1-263  IREFKNSESIGVPYPKNQPMRIYSSLWNADDWATRGGLVKTDWTQAPFTASYRNFNA.DACIWSSGAS......SCSSTTPSSTSTSGQWY
VvXTH30/1-273  IREFKNSESIGVPYPKNQPMRIYSSLWNADDWATRGGLVKTDWTQAPFNASYRNFNA.DACIWSSGAS......SCSSNTPTSTSTSTDWY
VvXTH31/1-297  VRVHTNMEKHGLPFPKDQPMGVYSSIWNADDWATQGGRVKTDWSHAPFIASYKGFEI.DACECPIAVAADDNAKRCSSSGE.KRYWWDEPT
VvXTH32/1-323  IRLFMRTKAIGNYFIT.SPMYVYGSIRDGSDWATDGGKQKVDYKYAPFTAAFSELIL.LGCPADPIDLKP..Y..CIDED.....PWEKKV
VvXTH33/1-252  IRLFMRIKAIGNYFIA.SPMYVHGSIWDTSDWTTDGGKQKVDYRYGPFTATSS..........................................
VvXTH34/1-285  IRVFANKEENGAPYPKTQAMGVQGSVWNADDWATQGGRVKTNWSHAPFVSTFRGFEI.DACELSPETD.DIASSKCGR..V.GEFWWDKPR
```

```
               200      210       220        230        240       250       260               270
VvXTH01/1-280  PTGIMPEQRSKMEKFRKTHMTYSYCYDRV.RYTV.PPSECVVNAQEAERLRRFDPTFGGRRHHHHGKRHQR........NRSSQGE..ATT
VvXTH02/1-314  FAKLSEQQRMGMEWARKKLMFYSYCKDSS.RFKV.LPPECKAK................................................
VvXTH03/1-308  FQDLDSLQYRRLAWVRQKYTIYNYCTDRV.RYPT.MPPECKRDRDV.............................................
VvXTH04/1-342  YSAITPLRRISMRKFRQKYMYYSYCYDTL.RYAT.PLPECVIIPSEKRRFKDTGRKFGGSHKKSSKRRSRV........PVATTTSDRQAA
VvXTH05/1-295  FSSLTSTQRRLFKWVRTHHLIYDYCQDNE.RFQNNLPKECFLPKY..............................................
VvXTH06/1-282  TQELDSYSRRRLRWVQKNFMIYNYCTDLK.RFPQGVPAECKRSRFNL............................................
VvXTH07/1-280  FQELDANSRRRLRWVQKNFMIYNYCTDYK.RFPQGLPPECKNSKV..............................................
VvXTH08/1-282  TQELDAYSRRRLRWVQKNFMIYNYCTDLK.RFPQGVPPECKHSRFNL............................................
VvXTH09/1-282  TQELDAYGRRRLRWVQKNFMIYNYCTDLK.RFPQGVPPECKHSRFNL............................................
VvXTH10/1-292  AGGLSQQQYSAMESVQKNYVVYDYCQDPK.RDHS.QIPEC...................................................
VvXTH11/1-281  FWKLDSTQQKAHENVRNKYMNYDYCSDTN.RFAV.RPKECTQ.................................................
VvXTH12/1-294  SGRLTGQQYRAMQWVQSRHMVYNYCSDNK.RDHS.LTPECWG.................................................
VvXTH13/1-301  AWHLSDSQKMDFAWVERNLVIYDYCKDTE.RYPQ.MLEECSLSPWD.............................................
VvXTH14/1-295  TQGLDAKSRRRLRWVQRYYMIYNYCTDMK.RFPEGLPPECRRSRSL.............................................
VvXTH15/1-291  NQGLDAKSRRKLQWVQKNYMIYNYCSDMK.RFPGGLPLECRH.................................................
VvXTH16/1-292  YSQLSYAKKGQMKWVRDNHMIYDYCKDTK.RFQGNMPPECFKPQF..............................................
VvXTH17/1-322  SQELDSTSQERMKWVQKNYMIYNYCSDTK.RFPQGLPPECTTTTIWDSPPWLSQVDFKDQQKMKWVEDNYMIYNYCADTGRFPQGLPTECT
VvXTH18/1-370  SQELDSTSQERMKWVQKNYMIYNYCTDTK.RFPQGLPPECTATTLS.............................................
VvXTH19/1-298  SQELDSTSQERMKWVQKNYMIYNYCADTK.RFPQGLPPECSATTMS.............................................
VvXTH20/1-286  YQELDSSSQEKLKWVQKNYMIYNYCTDTK.RFPQGLPPECTATNNS.............................................
VvXTH21/1-279  APELDARSQEKLMWVQRNYMIYNYCTDTK.RFPGGFPAECHATSNS.............................................
VvXTH22/1-313  KHELDSTSQARLRWVQKNYMIYNYCTDTK.RFPQSPPPECA..................................................
VvXTH23/1-297  SQELDSTSQERMKWVQKNYMIYNYCTDTK.RFPQGLPLECTATTTS.............................................
VvXTH24/1-298  SQELDSTSQERMKWVQKNYMIYNYCTDTK.RFPQGLPPECTATTTS.............................................
VvXTH25/1-297  SQELDSTSQERMKWVQKNYMIYNYCTDTK.RFPQGLSPECTATTTS.............................................
VvXTH26/1-297  SQELDSTSQERMTWVQKNYMIYNYCTDTKRFPQGLPPECTATTMS..............................................
VvXTH27/1-270  Q......FQNKTIYF.RVVTPYKY...................................................................
VvXTH28/1-297  SQELDSTSQERMKWVQKNYMIYNYCTDTK.RFPQGLPPECTATTTS.............................................
VvXTH29/1-263  SQELDSTSQERMKWVQKNYMIYNYCTDTK.RVPQGLPPECTAATMS.............................................
VvXTH30/1-273  SQEIDSTSQERMKWVQKNYMIYNYCTDTE.RFPQGLPPECTAATTS.............................................
VvXTH31/1-297  LAELNLHQSHQLLWVRANHMVYDYCTDSA.RFPA.TPVECQHHRH..............................................
VvXTH32/1-323  PTGLTRKEITQMRRFRHKFMTYSYCFDKE.RYPV.TPPECFVDGREMEPPQNFDPVFGGDPVEK...........................
VvXTH33/1-252  ...........................................................................................
VvXTH34/1-285  MSGLNRHKSHQLKWVRRRHLVYDYCMDSG.RFYE.MPRECIV.................................................
```

```
VvXTH01/1-280  I.
VvXTH02/1-314  ..
VvXTH03/1-308  ..
VvXTH04/1-342  A.
VvXTH05/1-295  ..
VvXTH06/1-282  ..
VvXTH07/1-280  ..
VvXTH08/1-282  ..
VvXTH09/1-282  ..
VvXTH10/1-292  ..
VvXTH11/1-281  ..
VvXTH12/1-294  ..
VvXTH13/1-301  ..
VvXTH14/1-295  ..
VvXTH15/1-291  ..
VvXTH16/1-292  ..
VvXTH17/1-322  VT
VvXTH18/1-370  ..
VvXTH19/1-298  ..
VvXTH20/1-286  ..
VvXTH21/1-279  ..
VvXTH22/1-313  ..
VvXTH23/1-297  ..
VvXTH24/1-298  ..
VvXTH25/1-297  ..
VvXTH26/1-297  ..
VvXTH27/1-270  ..
VvXTH28/1-297  ..
VvXTH29/1-263  ..
VvXTH30/1-273  ..
VvXTH31/1-297  ..
VvXTH32/1-323  ..
VvXTH33/1-252  ..
VvXTH34/1-285  ..
```
