# Supplementary material for: Identification and expression analysis of xyloglucan endotransglucosylase/hydrolase (XTH) family in grapevine (Vitis vinifera L.)
Source: PeerJ. 2022 Jun 13;10:e13546. doi: 10.7717/peerj.13546 (PMC9202548; doi:10.7717/peerj.13546)
Supplement: Supplemental Information 2 [file peerj-10-13546-s002.docx]

Table S1 Ka/Ks analysis and duplication date estimated for grape duplicated *XTH* paralogs.

| Paralogous Pairs | Ks | Ka | Ka/Ks | Duplication Date (million years ago) | Duplicate Type |
| --- | --- | --- | --- | --- | --- |
| VvXTH6/7  VvXTH8/9  VvXTH14/15  VvXTH17/19  VvXTH17/20  VvXTH17/21  VvXTH17/22  VvXTH17/23  VvXTH17/24  VvXTH17/25  VvXTH17/26  VvXTH17/27  VvXTH17/28  VvXTH17/29  VvXTH17/30  VvXTH18/19  VvXTH18/20  VvXTH18/21  VvXTH18/23  VvXTH18/24  VvXTH18/25  VvXTH18/26  VvXTH18/27  VvXTH18/28  VvXTH18/29  VvXTH18/30  VvXTH19/20  VvXTH19/21  VvXTH19/22  VvXTH19/23  VvXTH19/24  VvXTH19/25  VvXTH19/26  VvXTH19/27  VvXTH19/28  VvXTH19/29  VvXTH19/30  VvXTH20/21  VvXTH20/22  VvXTH20/23  VvXTH20/24  VvXTH20/25  VvXTH20/26  VvXTH20/28  VvXTH20/29  VvXTH21/25  VvXTH21/26  VvXTH22/23  VvXTH22/24  VvXTH22/25  VvXTH22/26  VvXTH22/28  VvXTH22/29  VvXTH23/24  VvXTH23/25  VvXTH23/26  VvXTH23/27  VvXTH23/28  VvXTH23/29  VvXTH23/30  VvXTH24/25  VvXTH24/26  VvXTH24/27  VvXTH24/28  VvXTH24/29  VvXTH24/30  VvXTH25/26  VvXTH25/27  VvXTH25/28  VvXTH25/29  VvXTH25/30  VvXTH26/27  VvXTH26/28  VvXTH26/29  VvXTH26/30  VvXTH27/28  VvXTH27/29  VvXTH27/30  VvXTH28/29  VvXTH28/30  VvXTH29/30 | 0.6337  0.0378  0.6800  0.3648  0.5472  0.9016  0.9349  0.5540  0.2524  0.4511  0.5264  0.3995  0.4385  0.3255  0.1639  0.5896  0.5519  0.9116  0.2818  0.4850  0.3015  0.3533  0.6340  0.2782  0.1887  0.4315  0.4352  0.8721  0.9149  0.4881  0.2816  0.4513  0.5087  0.3583  0.4346  0.5210  0.3261  0.9552  0.8772  0.4775  0.4918  0.4918  0.5420  0.4353  0.5107  1.0190  1.0022  0.8065  0.7936  0.7619  0.7926  0.7213  0.7950  0.5137  0.1931  0.1698  0.6678  0.1646  0.4012  0.5092  0.5625  0.5278  0.1977  0.5429  0.3625  0.1833  0.2173  0.6243  0.0863  0.3249  0.5296  0.7357  0.1638  0.4227  0.5396  0.6331  0.5312  0.3299  0.3091  0.4940  0.3508 | 0.0774  0.0062  0.0891  0.0260  0.0715  0.1462  0.0959  0.0414  0.0210  0.0352  0.0428  0.0886  0.0386  0.0545  0.0251  0.0662  0.0897  0.1524  0.0365  0.0672  0.0295  0.0348  0.1246  0.0311  0.0237  0.0749  0.0899  0.1523  0.1212  0.0655  0.0412  0.0565  0.0662  0.0910  0.0574  0.0524  0.0529  0.1486  0.1244  0.0747  0.1021  0.0762  0.0817  0.0755  0.0973  0.1445  0.1518  0.1276  0.1305  0.1304  0.1251  0.1267  0.1139  0.0828  0.0218  0.0211  0.1348  0.0211  0.0474  0.1003  0.0785  0.0783  0.0633  0.0772  0.0470  0.0330  0.0188  0.1360  0.0105  0.0455  0.0936  0.1466  0.0181  0.0466  0.0945  0.1374  0.1021  0.0946  0.0412  0.0912  0.0519 | 0.1221  0.1641  0.1311  0.0713  0.1307  0.1622  0.1026  0.0748  0.0834  0.0780  0.0813  0.2217  0.0880  0.1676  0.1529  0.1122  0.1626  0.1672  0.1296  0.1386  0.0978  0.0984  0.1966  0.1117  0.1256  0.1736  0.2065  0.1747  0.1325  0.1342  0.1465  0.1253  0.1301  0.2540  0.1322  0.1005  0.1623  0.1556  0.1418  0.1564  0.2076  0.1550  0.1507  0.1736  0.1905  0.1418  0.1515  0.1582  0.1644  0.1711  0.1578  0.1756  0.1432  0.1612  0.1131  0.1245  0.2019  0.1283  0.1183  0.1969  0.1395  0.1484  0.3203  0.1422  0.1296  0.1801  0.0866  0.2179  0.1213  0.1402  0.1767  0.1993  0.1104  0.1103  0.1752  0.2171  0.1922  0.2868  0.1333  0.1847  0.1480 | 48.75  2.91  52.31  28.06  42.09  69.36  71.92  42.62  19.41  34.70  40.49  30.73  33.73  25.04  12.61  45.36  42.45  70.12  21.68  37.31  23.20  27.18  48.77  21.40  14.51  33.19  33.48  67.09  70.38  37.55  21.66  34.71  39.13  27.57  33.43  40.08  25.08  73.48  67.47  36.73  37.83  37.83  41.69  33.48  39.29  78.39  77.09  62.04  61.04  58.61  60.97  55.49  61.16  39.51  14.86  13.06  51.37  12.66  30.86  39.17  43.27  40.60  15.21  41.76  27.88  14.10  16.71  48.02  6.64  24.99  40.74  56.59  12.60  32.52  41.51  48.70  40.86  25.38  23.78  38.00  26.98 | tandem  tandem  tandem  tandem  tandem  tandem  tandem  tandem  tandem  tandem  tandem  tandem  tandem  tandem  tandem  tandem  tandem  tandem  tandem  tandem  tandem  tandem  tandem  tandem  tandem  tandem  tandem  tandem  tandem  tandem  tandem  tandem  tandem  tandem  tandem  tandem  tandem  tandem  tandem  tandem  tandem  tandem  tandem  tandem  tandem  tandem  tandem  tandem  tandem  tandem  tandem  tandem  tandem  tandem  tandem  tandem  tandem  tandem  tandem  tandem  tandem  tandem  tandem  tandem  tandem  tandem  tandem  tandem  tandem  tandem  tandem  tandem  tandem  tandem  tandem  tandem  tandem  tandem  tandem  tandem  tandem |

Table S2 Primers used for qRT-PCR in this study.

| Name | Primer sequence |
| --- | --- |
| VvXTH-1-F | GCTGATTTGCTCTGTTCTTGGT |
| VvXTH-1-R | CGACGACAACTCCTGCTGTA |
| VvXTH-3-F | CCTGTGGAATGCGGATGACT |
| VvXTH-3-R | ATGGTGTATTTCTGGCGGACCC |
| VvHTX-4-F | CCGTGGAAGGGAAGAGAGGTAT |
| VvHTX-4-R | TGGCTTTGATGGGTAGTCGC |
| VvXTH-5-F | GCCCATTCATAGCCTCATTCAG |
| VvXTH-5-R | TGAGATGATGTGTCCTAACCCA |
| VvXTH-6-F | TCCCAAAGAACCAGCCCAT |
| VvXTH-6-R | GGACACCTTGAGGAAAGCGT |
| VvXTH-7-F | ACAAGGTCTCTGGCTCTGG |
| VvXTH-7-R | GACAGTGCCAGCAGAGTTG |
| VvHTX-10-F | GAAGGGAAGTGGAGATGGAAGA |
| VvHTX-10-R | TGCGGGAAATGTAGCGTCA |
| VvXTH-14-F | TGTGGAATGCTGATAACTGGGC |
| VvXTH-14-R | AGGACCAAATACAGGCGGCA |
| VvXTH-15-F | GGGAAAGGGAATAGAGAGCAGC |
| VvXTH-15-R | TCTTGTTGCCCAGTCATCAGC |
| VvXTH-17-F | CGGGCGTGCTAAGATACTCAAC |
| VvXTH-17-R | TGCTGTTCTCTGTTTCCCTTGC |
| VvXTH-20-F | TGGAATGCTGATGACTGGGC |
| VvXTH-20-R | CTGTGGGAAACGCTTTGTGTC |
| VvXTH-31-F | ATGCCTGTGAGTGCCCAATA |
| VvXTH-31-R | GGTGGCTCTGGTGTAGGTTC |
| VvXTH-32-F | CCACCACCCGTTTCCAATA |
| VvXTH-32-R | TTCGCTTGTCTTTGCCCA |
| VvXTH-34-F | CACCCGTAACTTCCACACCTAC |
| VvXTH-34-R | CCTCATTGGCTGGTTCTTTGG |
| Vvβ-actin7-F | TCAGGAAGGACCTCTATGGC |
| Vvβ-actin7-R  Vvβ-actin101-F  Vvβ-actin101-R | CTGTGGACAATGGATGGACC  TACAATTCCATCATGAAGTGTGATG  TTAGAAGCACTTCCTGTGAACAATG |


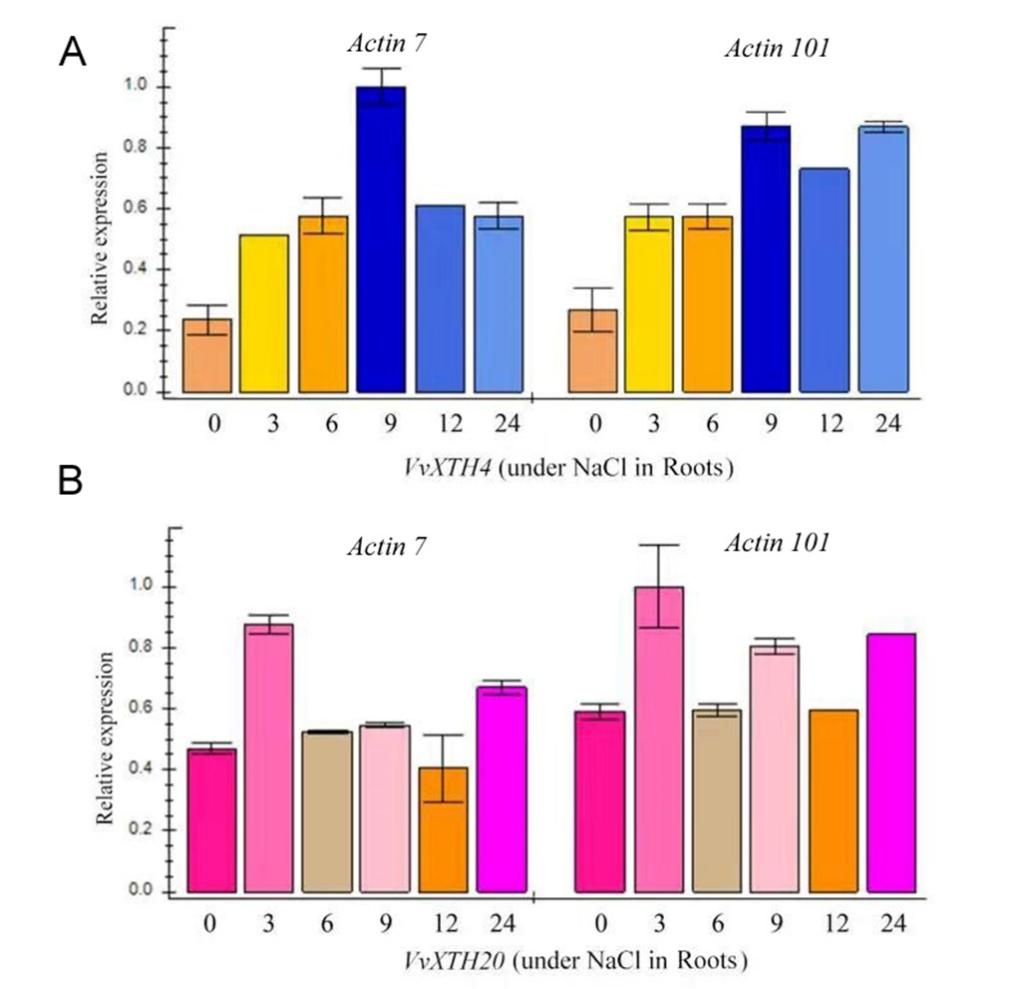


Figure S1 The relative expression of (A) *VvXTH4* and (B) *VvXTH20* in grapevine roots under salt stress normalize using *Vvβ-actin7* and *Vvβ-actin101*, respectively.


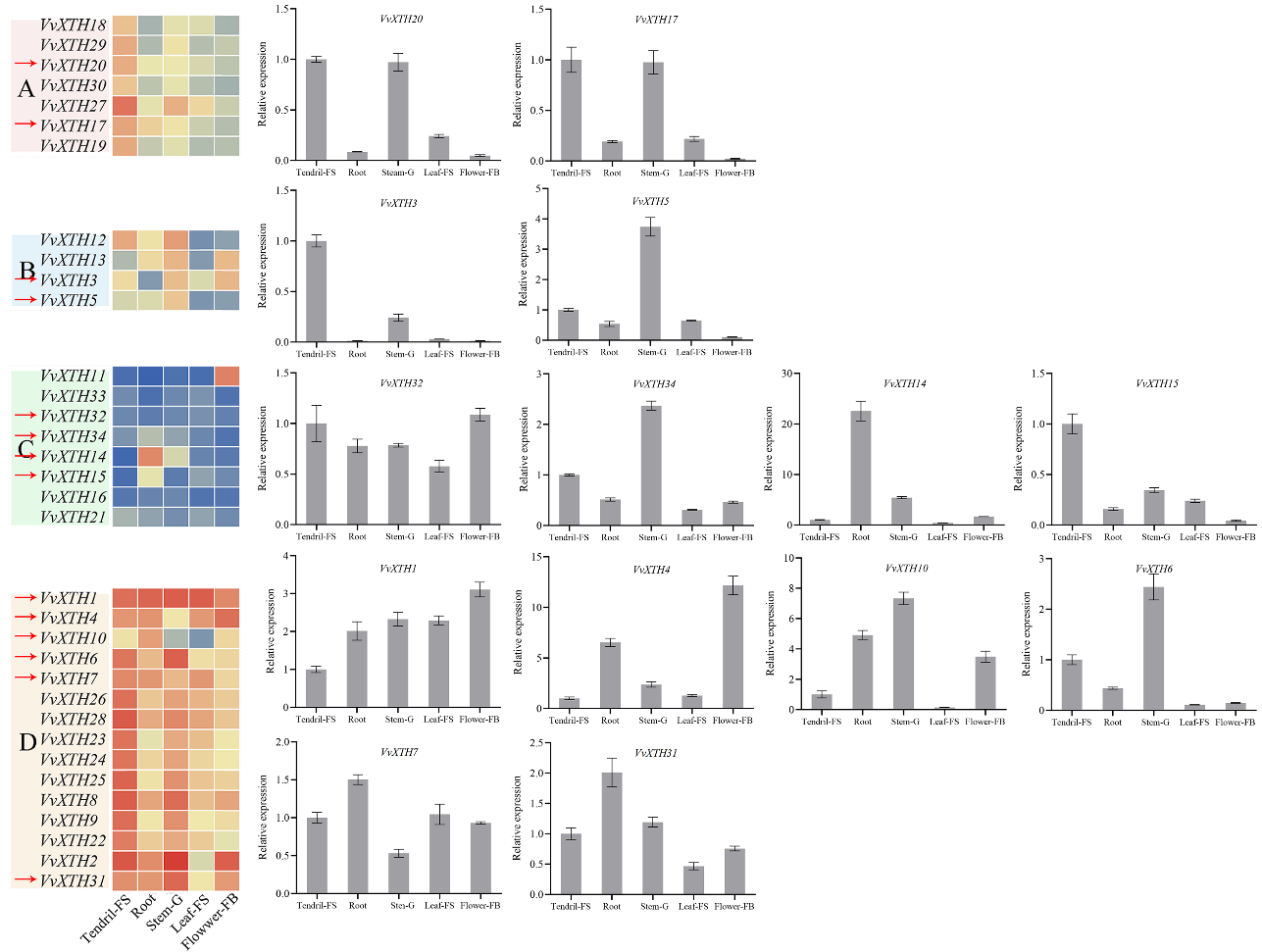


Figure S2 The comparison between quantitative qRT-PCR data and Microarray data.

The relative expressions of 14 selected *VvXTHs* were performed with qRT-PCR. The relative expression of tendril-FS was set up as 1. The y-axis indicates the folds of gene expression relative to tendril. Vertical bars indicate the standard error of the mean.

Note: Tendril-FS: A pool of mature-coiled tendrils collected at fruit set (berry size was ∼4 mm diameter); Root: Developing young roots; Stem-G: Stems collected starting from the second node from the tip; Leaf-FS: Mature leaves collected when the berry size was ∼4 mm diameter; Flower-FB: Flowers were collected at the beginning of flowering.


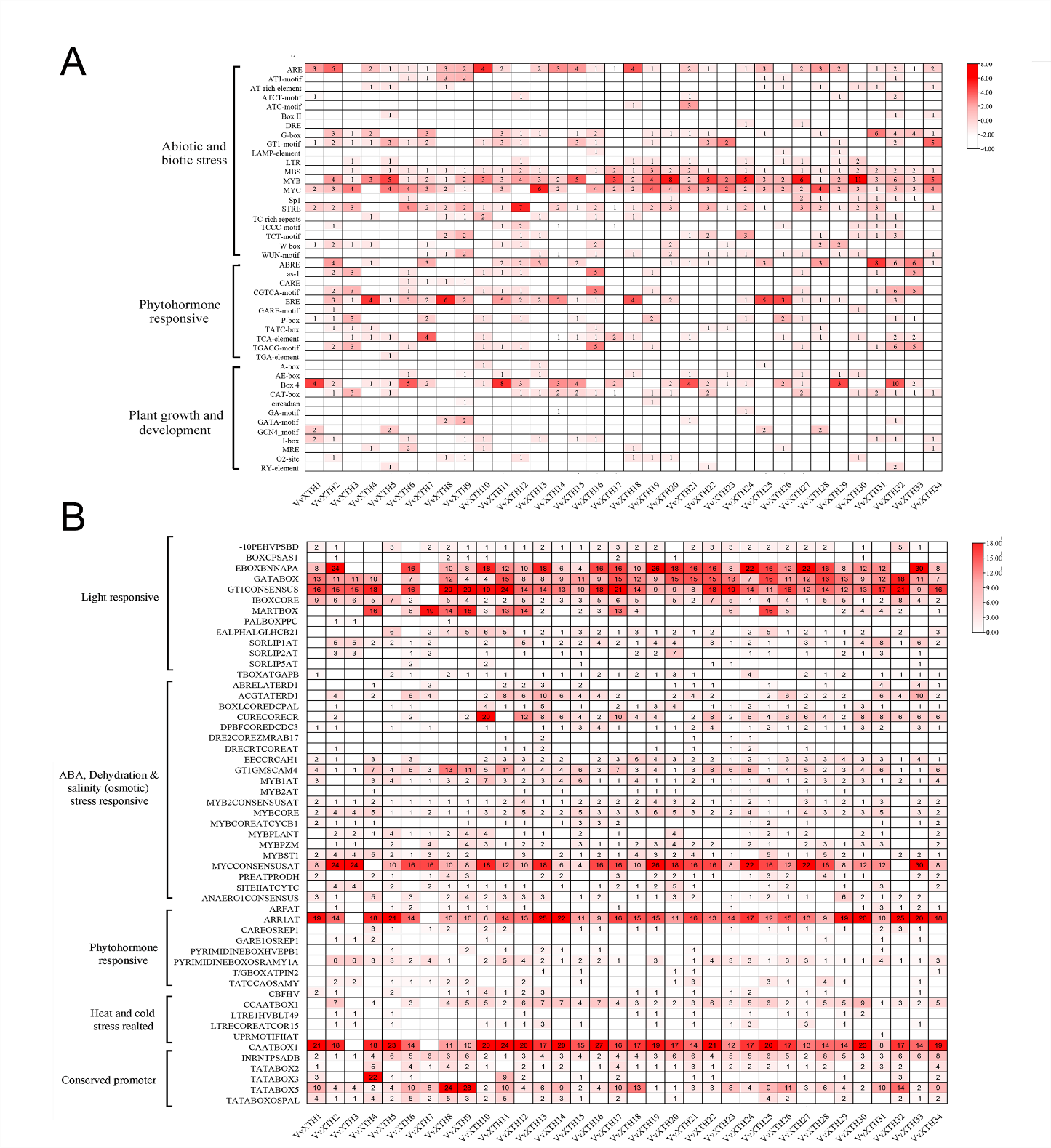


Figure S3 Heatmap of *cis*-element analysis

The prediction analysis was performed by using (A) plantCARE and (B) New PLACE. Columns represent *VvXTH* members, while rows show different *Cis*-element. The color intensity and number in the cells indicate the numbers of *cis*-element in these *VvXTH.*
